# Radiomics and machine learning for the diagnosis of pediatric cervical non-tuberculous mycobacterial lymphadenitis

**DOI:** 10.1038/s41598-022-06884-3

**Published:** 2022-02-22

**Authors:** Yarab Al Bulushi, Christine Saint-Martin, Nikesh Muthukrishnan, Farhad Maleki, Caroline Reinhold, Reza Forghani

**Affiliations:** 1 Augmented Intelligence and Precision Health Laboratory (AIPHL), Department of Radiology and the Research Institute of McGill University Health Centre, 5252 Boulevard de Maisonneuve O, Montréal, QC H4A 3S9 Canada; 2 Department of Radiology, McGill University Health Centre, 1001 Decarie Blvd, Montreal, QC H4A 3J1 Canada; 3 Radiomics and Augmented Intelligence Laboratory (RAIL), Department of Radiology and Division of Medical Physics, University of Florida, PO Box 100374, Gainesville, FL 32610-0374 USA; 4 Present Address: Department of Radiology, Stanford University, Stanford, CA 94305 USA

**Keywords:** Cancer imaging, Paediatric research, Brain imaging, Diagnostic markers, Computed tomography

## Abstract

Non-tuberculous mycobacterial (NTM) infection is an emerging infectious entity that often presents as lymphadenitis in the pediatric age group. Current practice involves invasive testing and excisional biopsy to diagnose NTM lymphadenitis. In this study, we performed a retrospective analysis of 249 lymph nodes selected from 143 CT scans of pediatric patients presenting with lymphadenopathy at the Montreal Children’s Hospital between 2005 and 2018. A Random Forest classifier was trained on the ten most discriminative features from a set of 1231 radiomic features. The model classifying nodes as pyogenic, NTM, reactive, or proliferative lymphadenopathy achieved an accuracy of 72%, a precision of 68%, and a recall of 70%. Between NTM and all other causes of lymphadenopathy, the model achieved an area under the curve (AUC) of 89%. Between NTM and pyogenic lymphadenitis, the model achieved an AUC of 90%. Between NTM and the reactive and proliferative lymphadenopathy groups, the model achieved an AUC of 93%. These results indicate that radiomics can achieve a high accuracy for classification of NTM lymphadenitis. Such a non-invasive highly accurate diagnostic approach has the potential to reduce the need for invasive procedures in the pediatric population.

## Introduction

Non-tuberculous mycobacteria (NTM) constitute an emerging infectious entity with increasing annual incidence in the pediatric and adult age groups^1^. In pediatric patients, NTM usually manifests as lymphadenitis, which is most often of the cervical lymph nodes^2–6^. The most common age group affected by NTM lymphadenitis is children of 1–5 years of age, and the disease is often indolent^1,7–10^. The annual incidence of NTM lymphadenitis has been reported to reach as high as 3.7 new cases per 100,000 children in children less than 5 years of age^1^. Recent reports have also suggested increased annual incidence in older children with reports of increased annual incidence at ages 11–14 years and new clusters of cases in patients aged 8–15^11,12^. Although the clinical picture of most NTM lymphadenitis cases is similar, it is not a straightforward diagnosis given the potential overlap with other infectious and non-infectious entities such as pyogenic lymphadenitis, tuberculous lymphadenitis, and proliferative lymphadenopathy^13,14^. The clinical presentation is typically of unilateral lymphadenopathy^5,10^. It is often painless and involves the submandibular and high anterior cervical lymph nodes^5,8,10^. Superficial skin extension has been reported in up to 15% of patients demonstrating spontaneous drainage through a sinus tract^5^. Prodromal symptoms are often absent and have been reported in less than 25% of patients^2–6,15^. The lack of specific clinical markers has led to relying on mostly invasive techniques for the diagnosis of NTM lymphadenitis in current clinical practice^5,13,15–25^.

Using current approaches, the diagnosis is usually delayed with up to 8–12 weeks of delay prior to specialist management^5^. It is imperative to exclude other possible organisms, such as TB, to guarantee proper management^6^. Nonetheless, diagnostic approaches such as the Tuberculin Skin Test (TST), Fine Needle Aspiration, or excisional biopsy have limitations in terms of sensitivity and specificity as well as the inadvertent complications of invasive testing^5,6,18,21,22^. The role of Purified Protein Derivative (PPD) has been evaluated with variable reported sensitivity of 5–50% for NTM and only improved role if cut off values are lowered which creates confusion in patients where prior TB exposure happened^4,6,10,13,26,27^. Fine Needle Aspiration (FNA) culture yield is reported at approximately 46%, with the predominant role of FNA in cases where malignancy is suspected^13,28^. A 5 year retrospective analysis indicated that as many as 41% of presumed NTM patients are treated with an unproven diagnosis^3^. Even following excisional biopsy and despite 100% specificity, the culture sensitivity has been reported to be around 41.8%, and the PCR sensitivity is reported to be 71.6%^28^. Acid-fast Bacilli (AFB) stains have also been utilized with sensitivity in the range of 46–85% and specificity in the range of 80–100%^28,29^.

**Figure 1:**
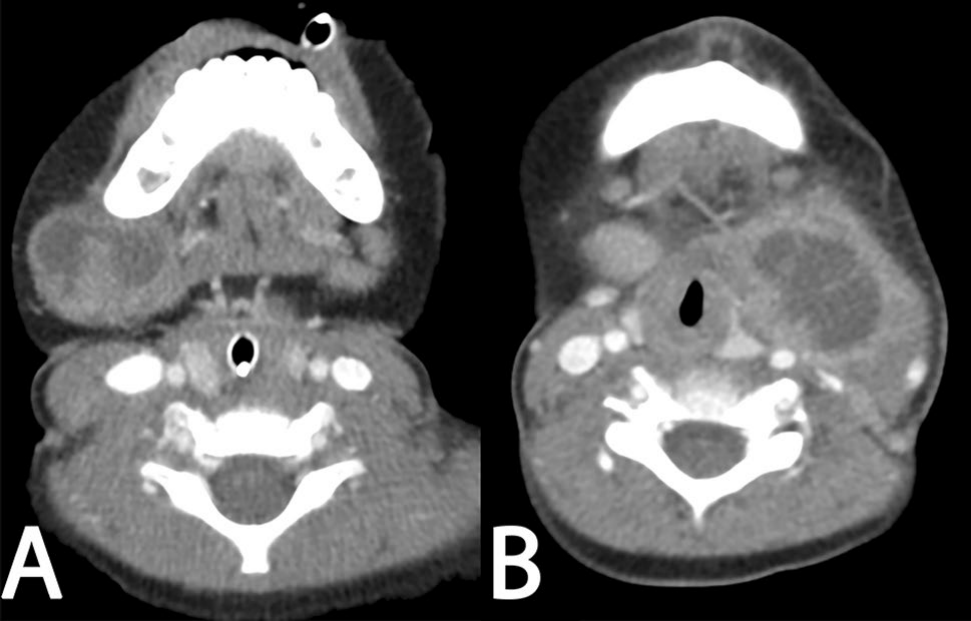
An example of a NTM Lymphadenitis diagnosis (**A**) and a pyogenic lymphadentis (**B**).

Certain signs have been proposed as potential imaging markers of NTM lymphadenitis. On limited case series, findings such as asymmetric lymphadenopathy, peripherally enhancing centrally cystic nodes, and often minimal surrounding inflammation with overlying skin thickening have been detected in the majority of reported cases of NTM lymphadenitis on cross-sectional imaging^16,24^. NTM lymphadenitis has also been associated with hypoechogenicity and intranodal liquefaction on ultrasound^19^. These imaging findings are not considered specific; therefore, diagnostic models rely on the clinical and invasive test results^5,16–19,21–25^. More reliable and reproducible noninvasive image-based biomarkers that can increase accuracy for the diagnosis of NTM lymphadenitis would therefore be of great interest. Figure 1 illustrates an example of a visually difficult to distinguish NTM lymphadenitis from a pyogenic lymphadenitis.

Radiomics refers to the use of medical images as mineable data and high-throughput extraction of quantitative features from those images for analysis and clinical decision support^30–33^. Machine learning can be a powerful tool for constructing prediction algorithms using such extracted features^34–36^. Radiomics aims to analyze and extract various complex quantitative features, many of which may not necessarily be evident or used based on qualitative image analysis alone^30,37–44^. Several studies have demonstrated the potential utility of image-based radiomic biomarkers for the evaluation of different malignancies. Recently more publications discussing the utility of radiomics and machine learning for the evaluation of lymph nodes have demonstrated the utility of such approaches^33,45–57^. Lymph node radiomic features have been suggested to be highly predictive of malignant versus benign etiology^33,45,46,48,50,54,58^. One study carried out in the pediatric population reported a sensitivity of up to 82.4% and a specificity of 86.2%^48^. Radiomic markers were also reported to be of high accuracy in the differentiation of the etiologies of lymphadenopathy, including a reported sensitivity of 91% and specificity of 93% for identifying malignant versus benign lymphadenopathy^33^. Also, the combination of primary tumor radiomics and lymph node radiomic markers has been found to have a potential added value for the prediction of management outcome and prognosis in lung cancer, cervical cancer, and head and neck cancers^47,49,51,53,59^.

The aim of this study is to develop and evaluate a radiomics-based machine learning classifier for noninvasive distinction of non-tuberculous mycobacterial lymphadenitis from other forms of lymphadenopathy in a pediatric cohort.

## Results

The classifiers designed to distinguish NTM, reactive, and proliferative lymphadenopathy achieved an average accuracy of 72%, precision of 68%, recall of 70%, F1-score of 67%, and area under the curve of 90%, when applied to samples in the test set. We observed no improvement in performance for the model when using 3- and 5-milimeter extensions of the contours. Table 1 shows the results for this model.

**Table 1:** The performance of the classifier for distinguishing non-tuberculous mycobacterial, reactive, or proliferative lymphadenopathy.

	Precision	Recall	F1	Accuracy	AUC
Contours-original	0.68 (0.08)	0.70 (0.09)	0.67 (0.08)	0.72 (0.07)	0.90 (0.04)
Contours-3 mm extension	0.64 (0.09)	0.66 (0.10)	0.63 (0.09)	0.69 (0.08)	0.87 (0.05)
Contours-5 mm extension	0.64 (0.09)	0.66 (0.10)	0.63 (0.09)	0.69 (0.08)	0.87 (0.05)

Reported results indicate the mean and standard deviation (in brackets) across 100 runs.

For distinction of NTM lymphadenitis from all other causes of lymphadenopathy, on the test set, the model achieved a precision of 65%, recall of 80%, accuracy of 82%, NPV of 91%, and area under the curve of 89%. The model performance from training on the extended contours are presented in Table 2. The ROC curve resulting from the original contours and the 3 mm extended contours were not found to be significantly different with a *p* value of 0.2114. Similar results were achieved when comparing the ROC curves resulting from the model developed using the original contours and the model developed using 5-milimeter extended contours with a *p* value of 0.1906.

**Table 2:** The distinction of NTM lymphadenitis from other causes of lymphadenopathy.

	Precision	Recall	F1	Acc	NPV	AUC
Contours-original	0.65 (0.10)	0.8 (0.12)	0.71 (0.08)	0.82 (0.05)	0.91 (0.05)	0.89 (0.05)
Contours-3 mm extension	0.63 (0.12)	0.68 (0.12)	0.65 (0.10)	0.80 (0.06)	0.87 (0.05)	0.85 (0.06)
Contours-5 mm extension	0.55 (0.10)	0.69 (0.13)	0.61 (0.09)	0.76 (0.05)	0.87 (0.06)	0.81 (0.07)

Reported results indicate the mean and standard deviation (in brackets) across 100 runs.

For the distinction of NTM lymphadenitis from pyogenic lymphadenitis, on the test set, the model achieved a precision of 92%, recall of 92%, accuracy of 88%, NPV of 72%, and area under the curve of 90%. The model performance from training on the extended contours are presented in Table 3. The ROC curve resulting from the model developed using the original contours and the model developed using 3-milimeter extended contours had a *p* value of 0.3166. Also, comparison of the ROC curves for the model developed using the original contours and the model developed using 5-milimeter extended contours had a *p* value of 0.3131.

**Table 3:** The distinction of NTM lymphadenitis from pyogenic lymphadenopathy.

	Precision	Recall	F1	Acc	NPV	AUC
Contours-original	0.92 (0.07)	0.92 (0.08)	0.92 (0.06)	0.88 (0.09)	0.72 (0.25)	0.9 (0.14)
Contours-3 mm extension	0.89 (0.09)	0.85 (0.10)	0.87 (0.07)	0.80 (0.10)	0.56 (0.26)	0.84 (0.13)
Contours-5 mm extension	0.89 (0.08)	0.81 (0.11)	0.84 (0.07)	0.77 (0.10)	0.48 (0.22)	0.78 (0.15)

Reported results indicate the mean and standard deviation (in brackets) across 100 runs.

For distinction of NTM lymphadenitis from reactive and proliferative lymphadenopathy, on the test set, the model achieved a precision of 75%, recall of 83%, accuracy of 85%, NPV of 91%, and area under the curve of 93%. The model performance from training on the extended contours are presented in Table 4. The comparison of the ROC resulting from the model developed using the original contours and the model developed using the 3-milimeter extended contours led to a *p* value of 0.0333. The comparison of the ROC curves resulting from the model developed using original contours and the model developed using the 5-milimeter extended contours led to a *p* value of 0.0126.

**Table 4:** The distinction of NTM lymphadenitis from reactive and proliferative lymphadenopathy.

	Precision	Recall	F1	Acc	NPV	AUC
Contours-original	0.75 (0.11)	0.83 (0.12)	0.78 (0.08)	0.85 (0.05)	0.91 (0.06)	0.93 (0.04)
Contours-3 mm extension	0.69 (0.13)	0.72 (0.12)	0.70 (0.10)	0.80 (0.06)	0.86 (0.06)	0.87 (0.06)
Contours-5 mm extension	0.63 (0.12)	0.73 (0.12)	0.67 (0.09)	0.78 (0.05)	0.86 (0.06)	0.83 (0.07)

Reported results indicate the mean and standard deviation (in brackets) across 100 runs.

## Discussion

In our study, we have demonstrated that radiomic features can distinguish NTM lymphadenitis from other causes of lymphadenopathy with accuracy of 82% and an area under the curve of 89%. The detailed analysis has also revealed that NTM can be distinguished from the most common causes of pediatric lymphadenopathy, reactive and proliferative etiologies, with accuracy of 85% and an area under the curve of 93%. One of the most challenging clinical and radiological assessments is to distinguish NTM lymphadenitis from pyogenic lymphadenitis. The latter can be potentially treated noninvasively in contrast to the surgical excision required for NTM lymphadenitis. Although our sample size for pyogenic lymphadenitis was limited, the model has achieved accuracy of 88% and an area under the curve of 90%.

The prevailing lack of specific imaging markers and shortcomings of the invasive diagnostic techniques for NTM lymphadenitis highlights the need for additional noninvasive diagnostic methods for nodal evaluation. Imaging has been frequently reported as a tool for pre-operative planning rather than diagnosis^5,6^. A report of ultrasound imaging features of NTM lymphadenitis found several prevalent findings pertaining to NTM such as decreased nodal echogenicity in 100% of the cases, central cystic changes in 92% of the patients and unilateral involvement in all but two of the reported cases. The study, however, did not compare the results of such findings to other entities of cervical lymphadenopathy and these signs remain non-specific^19^. Other imaging findings that have been described in several reports are the presence of ring-enhancing lymphadenitis with central heterogeneity or necrosis. However, these are also non-specific and can be seen in pyogenic lymphadenitis or metastatic lymphadenopathy^16,18,22–24,60,61^. On CT, it has been reported that in up to 90% of patients with NTM, there is a relative lack of moderate or severe surrounding soft tissue stranding, but this can also be seen in other entities such as other indolent infections or proliferative lymphadenopathy^16,17,24^. Cutaneous extension has been found to represent a more specific sign, present in 10 out of 12 patients by Robson et.al. and in 4 out of 6 patients by Hazra et al.^18,24^ but require evaluation in larger cohorts.

Quantitative assessment of pediatric lymphadenopathy utilizing texture or radiomic features has not been extensively studied. One report of nodal texture analysis in pediatric patients found a sensitivity in the range of 82.4–88.8% and a specificity in the range of 72.4–86% for detecting malignant lymph nodes^48^. Although there have been studies using radiomics or texture analysis for evaluation of neoplastic cervical lymph nodes in adults^33,47,49,51,53,58,59^, to our knowledge, our study is the first to assess radiomic features of NTM lymphadenitis. In addition, our analysis includes comparisons with pyogenic lymphadenitis, reactive and proliferative lymphadenopathy with a much larger sample size. Our results, although preliminary, are very promising, suggesting an important potential role of radiomic analysis in evaluation of pediatric NTM. One advantage of the radiomic approach is the non-invasive nature and utilization of available imaging studies without the need for a special examination or an invasive procedure.

In addition to evaluating the performance of radiomic models based on features extracted from the lymph nodes, our study also includes an analysis of the effects of expanding the contours to include the immediate perinodal soft tissues, an approach that is not frequently or consistently evaluated in radiomic studies. The ROC curves comparison revealed only a significant difference with the extended contours for the model used to discriminate NTM from the reactive and proliferative groups. All other comparisons resulted in a non-statistically significant performance drop. Based on this series, there was not a significant or consistent improvement in performance and the overall observations demonstrated a negative trend of the impact on performance by extending the contours, suggesting that proper contouring is required for optimal model performance in the paradigm investigated.

This study has a few limitations. The principal limitation is the sample size, which is an inevitable issue when studying such an uncommon clinical entity, yet our study has the largest cohort of NTM lymphadenitis patients with CT scans in comparison to prior reports on this topic. Another limitation is the fact that all cases come from one center. Reproducing the results when applied at a different institution will help validate the findings. The data was obtained from two scanners and cases with minor artifacts were included. Both factors introduce some heterogeneity to the data set which could highlight some practically relevant strength to the findings and their future generalizability. Lastly, we did not evaluate performance and potential combined models using radiomics and clinical characteristics which has the potential to further improve diagnostic performance and an important topic for future investigations.

In summary, this preliminary investigation demonstrates high accuracy of radiomics for discriminating NTM from other etiologies of lymphadenopathy in pediatric patients. If validated in larger cohorts from diverse institutions, this approach could provide the basis of an important non-invasive clinical decision support tool for this patient population, potentially improving diagnostic accuracy and diminishing the need for invasive procedures.

## Material and methods

### Patients

This study was conducted in accordance with applicable legislation, Declaration of Helsinki, and the Tri-Council Policy Statement for Ethical Conduct for Research Involving Humans, as well as in respect of the requirements set out in the applicable standard institutional procedures of the Research Institute of the McGill University Health Centre Research Institute. Approval was obtained by the McGill University Health Centre Research Ethics Board (REB# 2018-4460). Patient informed consent was waived by the McGill University Health Centre Research Ethics Board due to the retrospective and minimal risk nature of the study and because it would be impracticable to obtain informed consent with waiver of informed consent.

**Figure 2:**
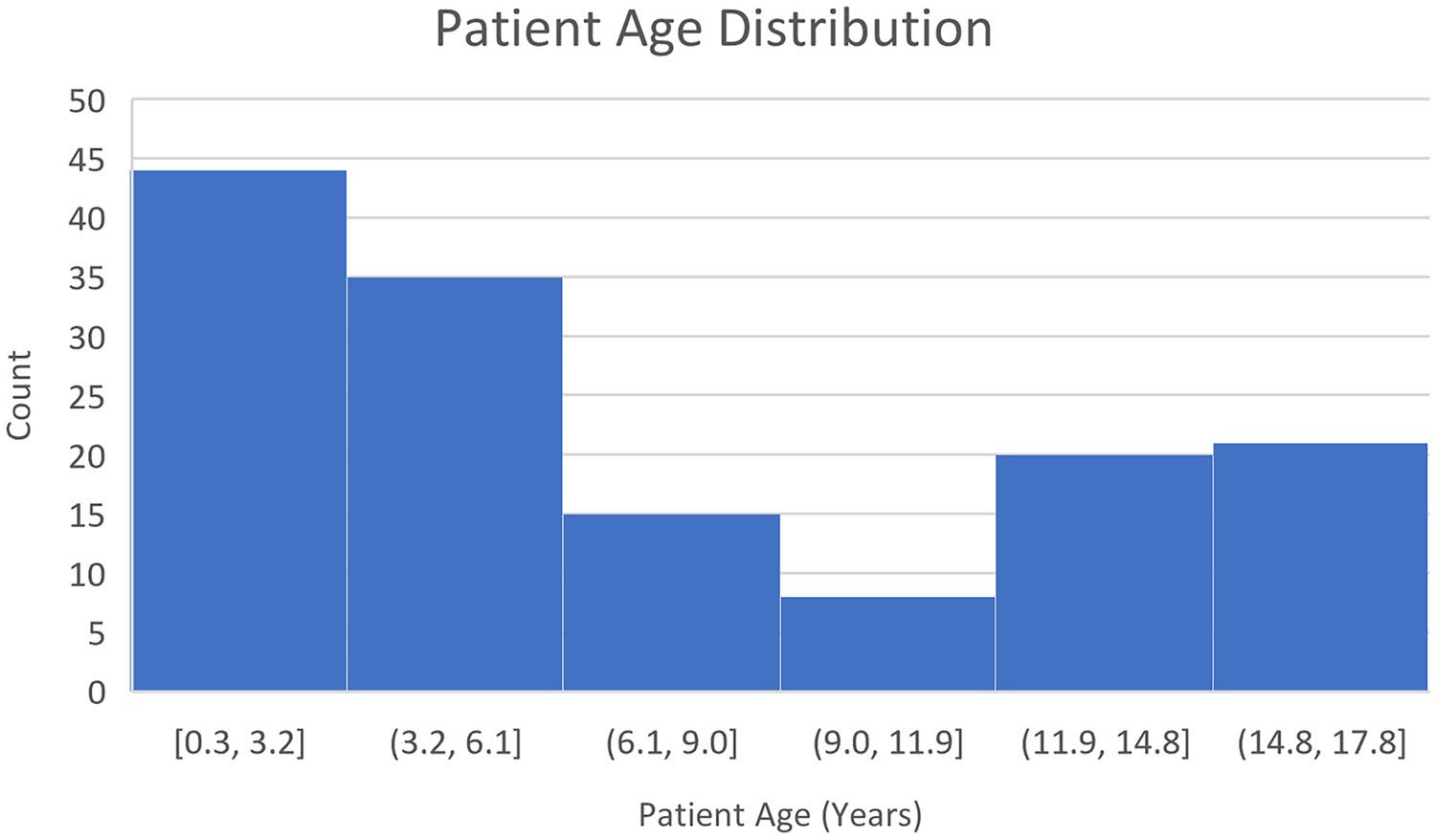
Patient age distribution.

We conducted a retrospective analysis of CT scans of pediatric patients aged 0–18 years who presented with lymphadenopathy to the Montreal Children’s Hospital between January 2005 and December 2018 (patient age distribution illustrated in Fig. 2). The studies were identified by a search of the PACS database in conjunction with the electronic health record (EHR). Out of 180 scans identified for potential analysis; 37 were excluded as follows: 8 scans were excluded as patients did not have a final definitive diagnosis, 6 scans were identified as post-treatment or follow up scans, 9 scans did not follow the standard protocol or had extensive artifacts limiting the assessment of nodal contours and 14 did not have any size significant lymph nodes as per the strict size criteria discussed below.

**Figure 3:**
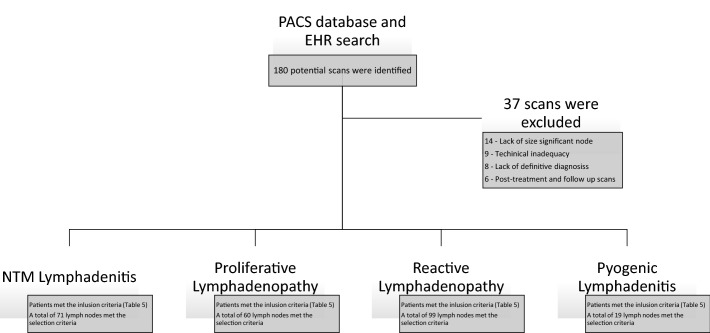
Study subjects—figure identifies the breakdown of the patient diagnosis. Specific selection criteria are described in Table 5.

A total of 249 lymph nodes from 143 scans of eligible patients met inclusion criteria (Table 5) for radiomic analysis. The patients were divided into four distinct groups, with one or multiple nodes analyzed from each patient. The groups were as follows (n = Total number of nodes): Proliferative nodes (n = 60, nodes proven to be involved by lymphoproliferative disorders), Reactive nodes (n = 99, reactive in the absence of known lymphoproliferative disorder, bacterial or mycobacterial nodal infection), NTM lymphadenitis (n = 71), Pyogenic lymphadenitis (n = 19, nodes proven to be involved by bacterial lymphadenitis). Figure 3 illustrates the distribution of patients and lymph nodes.

**Table 5:** Inclusion criteria.

Group	Scan parameter	Node and size criteria	Clinical/pathological confirmation
NTM	As per MCH standard CT protocol Field of view (FOV) includes the entire neck, particularly the involved node(s) Lack of artifacts that obscure more than 20% of nodal margins	10 mm shortest axial dimension	Scans included all cervical nodal stations Exclusion of other etiologies
Proliferative lymphadenopathy	As per MCH standard CT protocol Field of view (FOV) includes the entire neck, particularly the involved node(s) Lack of artifacts that obscure more than 20% of nodal margins	15 mm shortest axial dimension (Given that some nodal biopsies were performed for non-cervical lymphadenopathy we opted for a higher size cut off on this category)	Clinical and Histopathologic confirmation of the diagnosis
Reactive lymph nodes	As per MCH standard CT protocol Field of view (FOV) includes the entire neck, particularly the involved node(s) Lack of artifacts that obscure more than 20% of nodal margins	10 mm shortest axial dimension	Lack of active nodal infection or malignancy
Pyogenic lymphadenitis	As per MCH standard CT protocol Field of view (FOV) includes the entire neck, particularly the involved node(s) Lack of artifacts that obscure more than 20% of nodal margins	10 mm shortest axial dimension	Isolation of the causative organism from the involved node(s)

All proliferative lymph nodes were obtained from pathologically proven cases. The minimum acceptable size criterion for inclusion was kept at 15 mm to ensure that normal/reactive lymph nodes in a patient with known lymphoproliferative disorder are not included; a size criterion of 10 mm maximum short axis was used for all other categories (Supplementary Material A). We excluded lymphadenopathy in relation to head and neck or solid organ metastases due to lack of nodal pathology confirmation and heterogeneity of such a cohort of patients. The reactive nodes were obtained from scans performed for other non-neoplastic head and neck conditions with no evidence of clinical or radiological pathological nodal involvement. NTM lymphadenitis patients were included based on strict microbiologic and histologic criteria and/or confirmed histopathology combined with typical clinical context, including the microbiologic exclusion of other etiologies; subcategorization based on these two criteria was also performed. Pyogenic lymph nodes were all confirmed by isolation of the causative organism from the involved nodes as well as typical clinical presentation and course. Patients who underwent specific treatment prior to the imaging study, such as nodal incision and drainage or nodal excision, were excluded; follow-up scans after such procedures were also excluded. Patients without definitive pathological or serologic diagnosis and patients with overlapping clinical course were excluded as well.

### CT scan parameters

Patients were scanned using two Discovery CT750HD scanners (GE Healthcare) with 64 detectors, using a helical acquisition extending from the external auditory canal to the carina, after administration of IV contrast with a delay of 30 s. The studies were performed according to the Montreal Children’s Hospital standard protocol with axial reconstructions at a slice thickness of 2.5 mm. CT angiograms of the neck, non-contrast scans, scans with incomplete inclusion of the regions of interest, or scans with extensive artifacts that impair delineation of nodal margins were all excluded. A total of 143 scans were analyzed. For additional details on CT scan acquisition parameters, please refer to Supplementary Material B.

### Image analysis, node selection, and segmentation

Eligible scans were downloaded in DICOM format and de-identified prior to subsequent analysis. Each study was reviewed by an attending radiologist, and 1–4 nodes were selected for radiomic analysis based on the inclusion criteria described in Table 5. When applicable, the selected nodes corresponded to the subsequently biopsied or excised lymph nodes. Segmentation was performed using the open-source software 3D Slicer (Version 4.10.2)^62^. Prior to radiomic features extraction, manual contouring was first performed by a senior (fourth post-graduate year) diagnostic radiology resident (Y.A.B) and then reviewed (and modified if necessary) by a head and neck radiologist with 9 years (at the time of review) of post-fellowship experience (R.F.). These contours along with node-inclusive expanded contour margins (by 3 mm and by 5 mm) were exported from each selected lymph node for radiomic analysis.

### Radiomics analysis and machine learning classifier development

**Figure 4:**
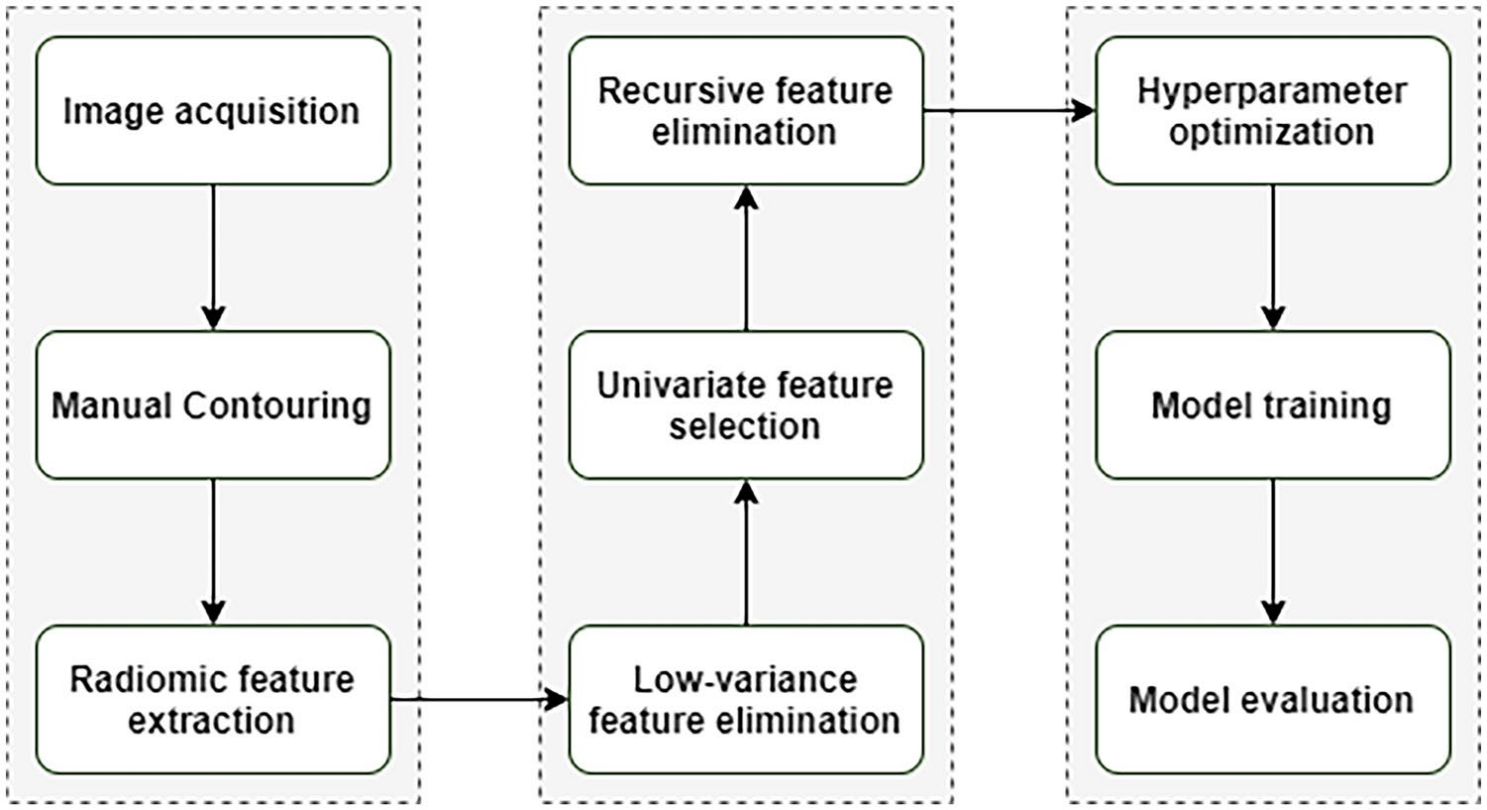
The pipeline for image data analysis—figure described the pipeline used to extract the imaging features as well as modeling steps.

Radiomic feature extraction for the primary contour was performed using the 3D Slicer integrated Pyradiomics extension^63^. This led to a 1231-dimensional numerical feature vector for each node, including first-order, second-order, and texture features. For an unbiased assessment of model performance, nodes from 20% of patients were selected for model evaluation (testing) and the rest of the nodes for model training. This was accomplished by a random assignment of nodes for each patient to either training set or test set. Since using a large number of features for model building could potentially lead to model overfitting^35^, the following steps were taken—using data in the training set—to reduce data dimensionality: First, all features with a zero variance were filtered. Second, we used a univariate feature selection for each remaining feature. The top 100 features with the most significant scores, corresponding to the 100 smallest *p* values, were selected. This was performed to find the most discriminative features, i.e. features with a significant difference across categories. Finally, a recursive feature elimination approach using a support vector classifier (SVC) with a linear kernel was used to select the ten most discriminative features as measured by feature importance values. Using the ten selected features, a Random Forest (RF) classifier was trained using a nested cross-validation with 5-inner and 5-outter folds^34^. A grid search of hyper-parameters (including the number and max depth of trees) was conducted with the reduced features to optimize model performance. Then—to achieve a reliable estimate of the generalization error—the optimal model was evaluated using the test data, i.e. data not being used in feature selection and model training steps. Due to the stochastive nature of machine learning model development, we repeated this process to built 100 models to achieve statistically reliable results. Then we reported average sensitivity, specificity, accuracy, precision, negative predictive value (NPV), and the area under the receiver operative curve (AUC) as performance measures. Figure 4 illustrates the methodology for image analysis. Using this methodology, a classification model was built to distinguish NTM, reactive, and proliferative nodes. We also built three binary classifiers for the primary contours to distinguish NTM related lymphadenitis (1) from reactive, proliferative, and pyogenic lymphadenopathy combined; (2) from pyogenic lymphadenopathy, and (3) from reactive and proliferative lymphadenopathy. For all experiments, the same procedure was followed to develop models using 3- and 5-milimeter extended contours of the nodes as well as the original contours. The ROC curves between the performance of the original and extended contours were compared using the test proposed in DeLong et al. to verify any significant improvement^64^. The analysis was conducted using Scikit-learn package (version 0.22.1) in Python (version 3.7.6)^65,66^. Supplementary Material D, and E provide additional model details regarding hyper-parameter range, and train and test sample distribution, respectively.

## Supplementary Information

Supplementary Information.

## Data Availability

We have provided three excel sheets (ContoursOriginalForReviewers.csv, Contours3mmForReviewers.csv, and Contours5mmForReviewers.csv) containing the extracted radiomic features for the reviewers. These sheets contain all 249 nodes, the corresponding patient, diagnosis, and the 1231 pyradiomics features.
