# Supplementary material for: Radiomics and machine learning for the diagnosis of pediatric cervical non-tuberculous mycobacterial lymphadenitis
Source: Sci Rep. 2022 Feb 22;12:2962. doi: 10.1038/s41598-022-06884-3 (PMC8863781; doi:10.1038/s41598-022-06884-3)
Supplement: Supplementary file 1 — Supplementary Information. [file 41598_2022_6884_MOESM1_ESM.docx]

**Supplementary Material A – Lymph node sizes**

Table A1. Size distribution for each diagnosis

| **Labels** | **Average of Short Axis Diameter (mm)** | **Standard Deviation (mm)** |
| --- | --- | --- |
| **NTM** | 16.86948 | 5.766568 |
| **Pyogenic** | 21.372 | 5.491081 |
| **Proliferative** | 17.75044 | 3.303497 |
| **Reactive** | 13.24966 | 2.727705 |

**Supplementary Material B – CT scan protocols**

Table B1. MCH standard CT scan protocols and parameters

|  | **Protocol for 2 years old and below** | **Protocol for above 2 years** |
| --- | --- | --- |
| Slice Thickness | 2.5 mm, gap 2.5mm provided reformations are 1.25mm thick | 5mm, gap 5mm, provided reformations are 2.5mm thick |
| FOV | 320 mm | 320 mm |
| kVp | 120 | 120 |
| mA | Variable depending on body weight and use of ASIR (50 to 60mA) | Variable (50 to 85mA) adapted automatically to body weight and use of ASIR |
| Acquisition Details | 30 seconds post injection of contrast (2ml per kg Omnipaque 300* peripheral IV) | 30 seconds post injection of contrast (2ml per kg Omnipaque 300* peripheral IV) |

**Supplementary Material C – Lymph Node Segmentation**

Figure C1. An example of manual segmentation of two enlarged right cervical lymph nodes. Axial CT images were initially utilized to contour each node individually (A). 3-dimensional rendering of the segmented lymph nodes are represented in green and yellow (B). 3D Segmentations of each individual lymph node were then exported for subsequent extraction of radiomic features.


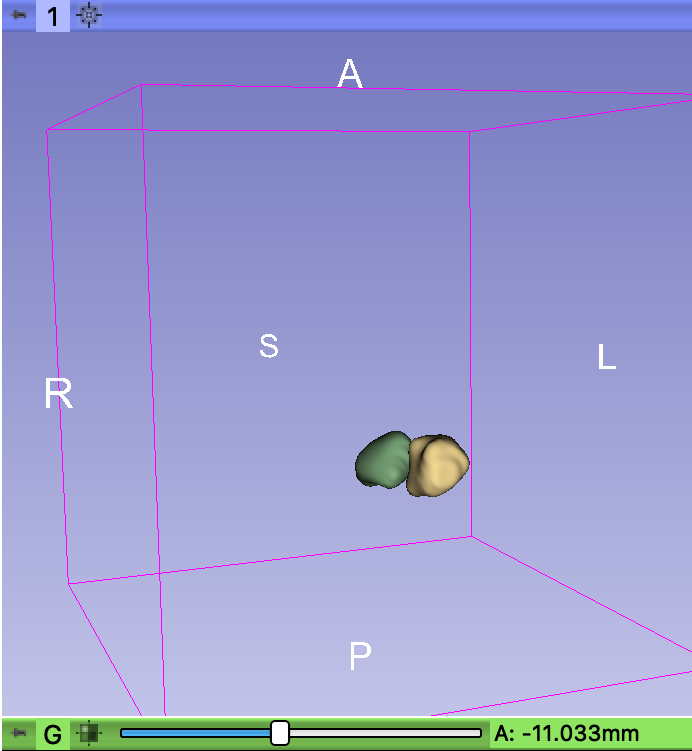

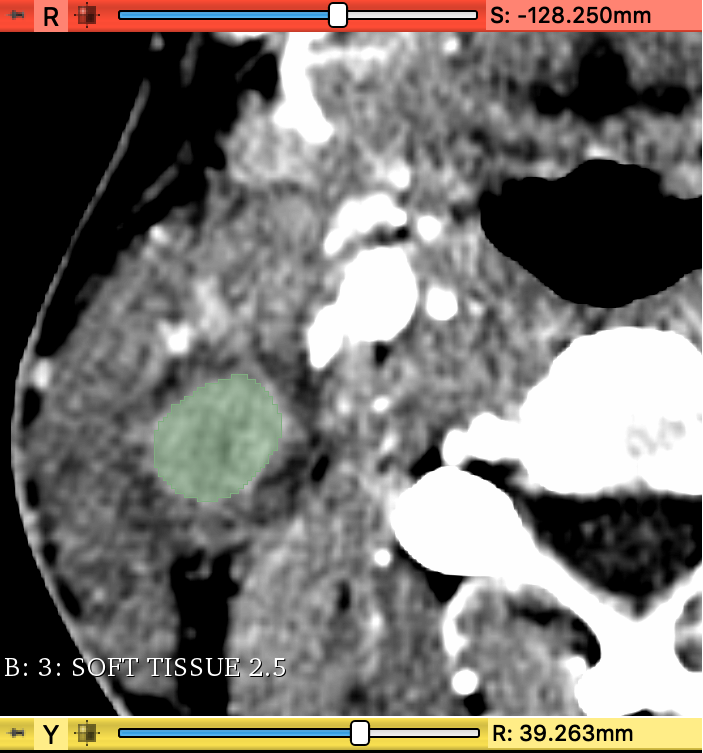


**Supplementary Material D: Hyperparameter search space**

| **Model Hyperparameter** | **Range of values** |
| --- | --- |
| N_estimators | 1000 |
| bootstrap | [True, False] |
| Max_depth | [10, 20, 25, 50, null] |
| Max_features | [‘auto’, ‘sqrt’] |
| Min_sample_leaf | [1, 2, 4] |
| Min_samples_split | [2, 5, 10] |
| Class_weight | Balanced |

**Supplementary Material E: Sample Distribution**

Table E1. Sample Distribution for Each Experiment

|  | | **NTM** | **Proliferative** | **Reactive** | **Pyogenic** |
| --- | --- | --- | --- | --- | --- |
| **NTM lymphadenitis from other** | **Train** | 46 | 35 | 79 | 14 |
|  | **Test** | 25 | 25 | 20 | 5 |
| **NTM lymphadenitis from pyogenic lymphadenopathy** | **Train** | 50 | 0 | 0 | 13 |
|  | **Test** | 21 | 0 | 0 | 6 |
| **NTM lymphadenitis from reactive, or proliferative lymphadenopathy** | **Train** | 57 | 45 | 57 | 0 |
|  | **Test** | 14 | 15 | 32 | 0 |
